# Disruption of Mitochondrial‐associated ER membranes by HIV‐1 tat protein contributes to premature brain aging

**DOI:** 10.1111/cns.14011

**Published:** 2022-11-23

**Authors:** Sterling P. Arjona, Charles N. S. Allen, Maryline Santerre, Scott Gross, Jonathan Soboloff, Rosemarie Booze, Bassel E. Sawaya

**Affiliations:** ^1^ Molecular Studies of Neurodegenerative Diseases Lab, Fels Cancer Institute for Personalized Medicine, Lewis Katz School of Medicine Temple University Philadelphia Pennsylvania USA; ^2^ Fels Cancer Institute for Personalized Medicine, Lewis Katz School of Medicine Temple University Philadelphia Pennsylvania USA; ^3^ Program of Behavioral Neuroscience, Department of Psychology University of South Carolina Columbia South Carolina USA; ^4^ Department of Cancer and Cellular Biology Lewis Katz School of Medicine, Temple University Philadelphia Pennsylvania USA; ^5^ Department of Neural Sciences Lewis Katz School of Medicine, Temple University Philadelphia Pennsylvania USA

**Keywords:** aging, HIV‐1‐tat, MAM‐tethering, memory impairment, mitochondria‐associated ER membranes, PTPIP51, VAPB

## Abstract

**Introduction:**

Mitochondrial‐associated ER membranes (MAMs) control many cellular functions, including calcium and lipid exchange, intracellular trafficking, and mitochondrial biogenesis. The disruption of these functions contributes to neurocognitive disorders, such as spatial memory impairment and premature brain aging. Using neuronal cells, we demonstrated that HIV‐1 Tat protein deregulates the mitochondria.

**Methods& Results:**

To determine the mechanisms, we used a neuronal cell line and showed that Tat‐induced changes in expression and interactions of both MAM‐associated proteins and MAM tethering proteins. The addition of HIV‐1 Tat protein alters expression levels of PTPIP51 and VAPB proteins in the MAM fraction but not the whole cell. Phosphorylation of PTPIP51 protein regulates its subcellular localization and function. We demonstrated that the Tat protein promotes PTPIP51 phosphorylation on tyrosine residues and prevents its binding to VAPB. Treatment of the cells with a kinase inhibitor restores the PTPIP51‐VAPB interaction and overcomes the effect of Tat.

**Conclusion:**

These results suggest that Tat disrupts the MAM, through the induction of PTPIP51 phosphorylation, leading to ROS accumulation, mitochondrial stress, and altered movement. Hence, we concluded that interfering in the MAM‐associated cellular pathways contributes to spatial memory impairment and premature brain aging often observed in HIV‐1‐infected patients.

## Graphical abstract

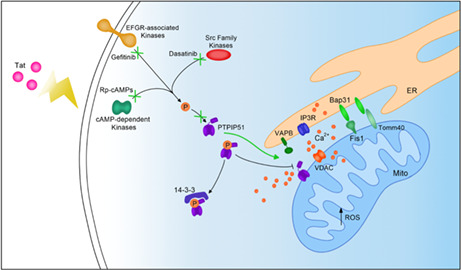

## 1 INTRODUCTION

Despite years of basic, translational, and clinical research, HIV‐1‐associated neurocognitive disorders (HAND) remain a challenge for people living with HIV‐1 (PLWH).^1^, ^2^ A recent meta‐analysis identified the prevalence of HAND to be about 44%.^3^ HAND symptoms include learning disability and spatial memory impairment. Therefore, it is crucial to determine the cellular and molecular players involved to develop a better therapy.

HIV‐1 does not infect neurons; however, the neurons contribute to the cognitive defects seen in HAND.^4^ Neuronal deregulation and persistent inflammation are caused by released viral proteins, such as Tat, from infected astrocytes, microglia, endothelial, and infiltrating macrophages.^5^ In addition to Tat, infected cells release other viral proteins like gp120, Tat, Nef, and Vpr, and proinflammatory toxins.^6^ The presence of Tat protein in the extracellular environment and neurons leads to increased calcium influx and reactive oxygen species (ROS), mitochondrial deregulation, and altered synaptic plasticity.^7^, ^8^, ^9^

Mitochondria‐associated endoplasmic reticulum (ER) membranes (MAMs) define the dynamic functional relationship between the ER and mitochondria.^10^ In recent years, MAMs have been the focus of multiple studies that elucidate their many functions. Mitochondria‐ER association is crucial for calcium and lipid transfer between the two organelles.^11^ Because of the role MAMs play in cellular homeostasis, their dysfunction leads to aging and senescence diseases.^12^, ^13^, ^14^, ^15^ MAMs also regulate cellular functions in neurons like postsynaptic energetics.^16^

HIV‐1 proteins cause ER stress and induce the unfolded protein response (UPR) in astrocytes and neurons.^17^ MAMs are partially responsible for regulating these ER stress and UPR signals, suggesting that they play a role in HAND pathology.^17^ Further, the ability of HIV‐1 Tat to alter calcium signaling and homeostasis, functions regulated by MAMs, supports the idea that MAMs contribute to HAND pathology.^9^, ^15^, ^18^, ^19^

Given the role of MAMs in neurodegeneration, the focus of the current investigation is to confirm MAMs' contribution to the development of HAND pathology using SH‐SY5Y and Lund human mesencephalic (LUHMES) cells.^9^, ^20^ The expected results study will bring us closer to elucidating a potential therapy directed against HAND.

## 2 METHODS

### 2.1 Cell Culture

SH‐SY5Y neuroblastoma cells were purchased from ATCC (CRL‐2266) and were maintained as described.^9^ Cells were differentiated with 10 μM retinoic acid for at least 4 days before treatment and subsequent experiments. LUHMES cells were purchased from ATCC (CRL‐2927) and Abm (T0284) and were maintained and differentiated as previously described.^21^, ^22^ SH‐SY5Y cells were used for characterizing the global protein expression and MAM fractionation because these cells have been previously used by our lab and others for studying the mechanisms involved in HAND. LUHMES were used because they are not cancer cell line and do not exhibit cancer‐like metabolic phenotypes. LUHMES have also been used to study other neurodegenerative diseases.

### 2.2 Tat Treatment

Recombinant Tat protein was obtained from the NIH‐ AIDS Reagent Program (HIV‐1 IIIB Tat Protein, ARP‐2222). The protein was reconstituted according to the datasheet in PBS containing 1 mg/ml BSA and 0.1 mM DTT. All experimental cells were treated with 100 ng/ml Tat or PBS with BSA and DTT (labeled control) for 24 h as indicated.

### 2.3 Proteomics

SH‐SY5Y cells were treated with Tat or PBS for 24 h and then collected with 0.25% trypsin and centrifuged at 4°C at 200x*g* for 5 min to form cell pellets. Mass spectrometry analysis was performed and analyzed at the Temple University Proteomics Facility.^9^ Heat maps were made using Microsoft Excel.

### 2.4 Western Blot

Cells were collected and lysed with radio‐immunoprecipitation assay (RIPA) buffer (25 mM Tris–HCl pH 7.6150 mM NaCl, 1%Triton X‐100, 0,1%SDS). Western blot analysis was performed using primary antibodies as indicated.^9^ Secondary antibodies used to detect protein bands are: antimouse IgG‐HRP 1:10,000 (Advansta, R‐05071‐500) and antirabbit IgG‐HRP 1:10,000 (Advansta, R‐05072‐500). Densitometry of protein bands was determined using ImageJ software.

### 2.5 Antibodies and Dilutions for Western Blot

Anti‐VDAC 1:1000 (Cell Signaling, 4661), anti‐PTPIP51 1:1000 (Proteintech, 20,641‐1‐AP), anti‐VAPB 1:1000 (Invitrogen, MA5‐24348), anti‐Grp75 1:5000 (Proteintech, 14,887‐1‐AP), anti‐Bap31 1:1000 (Enzo Life Sciences, ALX‐804‐601‐C100), anti‐Fis1 1:1000 (GeneTex, GTX111010), anti‐Tomm40 1:1000 (Novus Biologicals, NBP2‐94075), anti‐IP3R‐I/II/III 1:500 (Santa Cruz Biotechnology, sc‐377,518), anti‐H3 0.5 μg/ml (GenScript, A01502), anti‐Actin 1:200 (Santa Cruz Biotechnology, sc‐8432).

### 2.6 Proximity Ligation Assays

LUHMES were differentiated for 1 day and then split onto glass coverslips coated with poly‐L‐ornithine and fibronectin as described^22^ and allowed to differentiate for at least three more days. Cells were then treated with Tat or PBS. After 24 h, cells were fixed in 4% formaldehyde in PBS for 20 min, then washed in PBS twice for 5 min. Next, the cells were permeabilized in PBS with 0.2% Triton X‐100 for 5 min and washed in PBST twice for 5 min each. Afterward, the cells were blocked in Duolink 1x blocking solution for 1 h at 37°C. Cells were probed for protein interactions with a Duolink in Situ PLA kit (Sigma‐Aldrich DUO92001, DUO92005, DUO92008) according to the manufacturer's instructions. Primary antibodies were diluted to 1:350 in Duolink Antibody Diluent. Anti‐pTyr 1:350 (Cell Signaling, 9411) was used to detect the phosphorylation of PTPIP51. Images were taken on a Leica EL6000 DMI3000 confocal microscope and analyzed using ImageJ.

### 2.7 Immunohistochemistry

HIV‐1 transgenic rat brain tissue was obtained from Pr. Rosemarie Booze (U. South Carolina). This animal model expresses an HIV‐1 provirus lacking gag‐ and pol‐ to render these animals noninfectious.^23^ The animals were housed and treated as previously described.^24^ Frozen brains were fixed and sectioned at Fox Chase Histopathology Facility. The tissue was stained with anti‐PTPIP51 and anti‐VAPB antibodies and counterstained with hematoxylin. Images were taken using Aperio ImageScope software and analyzed in ImageJ.

### 2.8 MAM Fractionation

SH‐SY5Y cells were differentiated for at least 4 days and then treated with Tat or PBS for 24 h as described above. Cells were collected and lysed in a sucrose homogenization buffer and subjected to fractionation.^25^, ^26^ MAM and cytosolic fractions were collected and subjected to Western blot analysis.

### 2.9 Seahorse Mito Stress Test

A Seahorse Mito Stress test measuring changes in oxygen consumption rate was conducted using a Seahorse XFe96 Analyzer (Agilent Technologies). LUHMES were differentiated for 5 days and then split onto an XFe96‐well microplate coated with poly‐L‐ornithine and fibronectin and allowed to grow overnight. The cells were then treated with Tat or PBS for 24 h. The Mito Stress test was performed as described^22^ and per the Agilent Technologies protocol.

### 2.10 Reactive Oxygen Species Measurement

RedoxSensor Red CC‐1 (R‐14060) and MitoTracker Green FM (M‐7514) were purchased from Fisher Scientific. LUHMES were differentiated for 1 day and then split onto glass coverslips and allowed to grow for 4 days. Afterward, cells were treated with Tat or PBS for 24 h as described above. Cells were incubated with 1 μM Redox Sensor Red CC‐1 and 1 μM MitoTracker Green FM at 37°C for 10 min. The cells were washed with PBS, the media was replaced, and allowed to sit for 20 min in a cell culture incubator. Cells were then imaged using a Leica EL6000 DMI3000 confocal microscope and images were analyzed using ImageJ.

### 2.11 Metabolomics for Glutamate and ATP and ADP Expression

LUHMES were differentiated as described above. On the last day of differentiation, a glucose‐free medium with D‐Glucose‐13C6 (Aldrich, 389,374‐250MG) was added to the cells for 24 h. The cells were then treated with 100 ng/ml Tat or PBS for 8 h. Polar metabolites were extracted in ice‐cold 80% methanol. Mass spectrometry was conducted at the Proteomics and Metabolomics Center‐Wistar Institute, Philadelphia.^27^

### 2.12 Glutamate Assay

LUHMES were differentiated and treated with PBS or 100 ng/ml Tat as described above. Cell medium (supernatant) was collected after 24 h and subjected to a colorimetric Glutamate Assay Kit (Cell BioLabs, MET‐5080) according to the manufacturer's instructions. Absorbance at 450 nm was read on a GloMax Multi Detection System plate reader (Promega) and analyzed in Prism 9 (GraphPad).

### 2.13 Calcium Labeling and Imaging

LUHMES were grown on glass coverslips coated in PLO and fibronectin and were then differentiated and treated with Tat or PBS as described above. On the day of the experiment, 2 μM Rhod‐2 AM (Invitrogen, R1245MP) and 0.5 M ci‐IP_3_/PM (Tocris, 6210) were added to the cells for 1 h. Afterwards, media was replaced by a calcium buffer (107 mM NaCl, 7.2 mM KCl, 1.2 mM MgCl_2_, 6H2O, 11.5 mM glucose, 20 mM HEPES‐NaOH, and 1 mM CaCl_2_, pH 7.2) containing 2 μM Fura‐2 AM (Invitrogen, F1221) and 0.5 M ci‐IP_3_/PM for 30 min. This solution was replaced by calcium buffer without Fura‐2 AM or ci‐IP_3_/PM for another 30 min. The cells were then imaged on a Leica microscope in a calcium‐free buffer (107 mM NaCl, 7.2 mM KCl, 1.2 mM MgCl_2_ 6H_2_O, 11.5 mM glucose, 20 mM HEPES‐NaOH, pH 7.2) for 2 min for a baseline reading of intracellular calcium levels. Next, 1 mM CaCl_2_ was added to the cells, and imaging was continued for another 2 min. Caged‐IP_3_ (ci‐IP_3_) was uncaged by exposure to UV light (λ395) for 20 s and imaging was continued for 2 min. Cells were imaged on a DMI 6000B fluorescence microscope (Leica Microsystems) as described.^28^ Fluorescence intensity was measured in arbitrary units (AU). The change in fluorescence (ΔAU) was calculated and analyzed using Prism 9 (GraphPad).

### 2.14 Kinase Inhibitors

Dasatinib (Cayman Chemical Company, 11,498) is an inhibitor for non‐receptor tyrosine kinases such as cAbl and members of the Src family. Gefitinib (Cayman Chemical Company, 13,166) inhibits EGFR and EGFR‐associated kinases. Rp‐cAMPs (Enzo Life Sciences, BML‐CN135‐0001) is an inhibitor for cAMP‐dependent protein kinases such as PKA. All three kinase inhibitors have been shown to modulate PTPIP51 phosphorylation and the ability for PTPIP51 to interact with other proteins.^29^ LUHMES were differentiated on glass coverslips coated in PLO and fibronectin and treated with Tat or PBS for 24 h as described above. Dasatinib (400 nM), Gefitinib (80 μM), and Rp‐cAMPs (40 μM) were added to the cells at the same time and allowed to incubate for 4 h. The cells were then washed in PBS and fixed in 4% formaldehyde in PBS for 20 min. PLA was conducted as described above using anti‐pTyr 1:350 (Cell Signaling, 9411) and anti‐PTPIP51 (Proteintech, 20,641‐1‐AP) primary antibodies. Images were taken on a Leica EL6000 DMI3000 confocal microscope and analyzed using ImageJ.

### 2.15 Statistics

Statistical analysis was conducted in Prism 9 (GraphPad) where normality was determined by the Shapiro–Wilk test. Student's t‐tests were conducted when the data were normally distributed, and Mann–Whitney tests were conducted when the data exhibited a non‐parametric distribution. Results were considered statistically significant if *p* < 0.05.

## 3 RESULTS

### 3.1 Tat protein disrupts calcium homeostasis and mitochondrial energy in LUHMES

**FIGURE 1:**
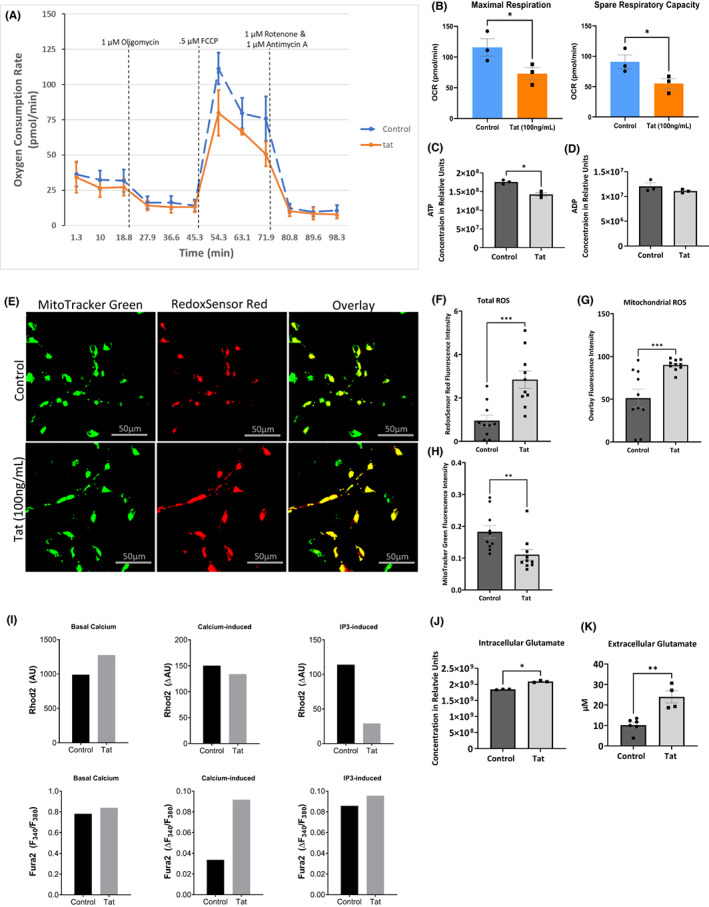
Tat deregulates mitochondrial energy and increases ROS. (A) Graph illustrating a Seahorse Mito Stress Test measuring oxygen consumption rate (OCR) in control LUHMES and those treated with Tat. (B) Representative bar graphs showing OCR associated with maximal respiration and spare respiratory capacity. (C, D) Bar graphs showing intracellular ATP, and ADP, respectively from metabolomics analysis of control LUHMES and cells treated with Tat in relative concentration units. (E) Representative images of RedoxSensor Red and MitoTracker Green staining for ROS in control LUHMES and those treated with Tat. (F, G and H) Quantification of total ROS, mitochondrial ROS, and mitochondrial number, respectively (represented as fluorescence intensity of MitoTracker Green stain) represented as bar graphs. (I) Mitochondrial (Rhod2) and cytosolic (Fura2) calcium levels in live control LUHMES and those treated with Tat at basal levels, in response to additional extracellular calcium, and uncaged IP_3_. Results are from a single experiment and represented as arbitrary units (AU) and delta arbitrary units (ΔAU) in bar graphs. (J) Glutamate concentration in LUHMES in the absence and presence of Tat as obtained from metabolomics. (K) Extracellular glutamate concentration in LUHMES in the absence and presence of Tat using a glutamate assay and cell supernatant. (N.S. not significant). Results were judged statistically significant if *p* < 0.05 by analysis of variance. (*p < 0.05; **p < 0.01; ***p < 0.001). Error bars are shown as S.E.M.

Previous studies reported the ability of HIV‐1 Tat protein to increase calcium influx and cause loss of mitochondrial energy. However, none of these studies used LUHMES cells; therefore, we assessed Tat functions in these cells. First, we determined whether the addition of Tat protein affects mitochondrial bioenergetics. We measured the oxygen consumption rate (OCR) of LUHMES in the presence of Tat using the Seahorse Mito Stress Test (Figure 1A). The addition of FCCP, an uncoupling agent, revealed decreased maximal respiration in the presence of Tat (Figure 1B, left). The spare respiratory capacity, the difference between maximal and basal respiration, was also reduced (Figure 1B, right). Because both maximal respiration and spare respiratory capacity indicate the cells' ability to function in a high energy‐demanding state, we concluded that Tat protein disturbs cellular bioenergetics, which corroborates our previous data using cells treated with gp120 protein.^22^

Mitochondria energy dynamics are known to be regulated by MAMs^30^; therefore, we measured the concentration of ATP and ADP in LUHMES treated with Tat or PBS using mass spectrometry for metabolites (Figure 1C, D). The addition of Tat significantly decreases ATP levels in these cells, thus supporting the reduction in OCR seen in the Seahorse Mito Stress Test experiment.

MAMs regulate mitochondrial reactive oxygen species (ROS)^13^; hence, we measured ROS levels in Tat‐treated LUHMES cells using the RedoxSensor Red dye (Figure 1E). RedoxSensor Red dye localizes to the cytosol, mitochondria, or lysosomes. In the presence of Tat, the total ROS levels, indicated by the RedoxSensor Red fluorescence intensity, were significantly elevated compared to control cells (Figure 1F). We also labeled mitochondria with a counterstain (MitoTracker Green), so the colocalization of the RedoxSensor Red and MitoTracker Green can be imaged and visualize redox potential in the mitochondria. The addition of Tat increased the colocalization of RedoxSensor (red) and MitoTracker (green), suggesting that mitochondrial ROS also increased in these cells (Figure 1G).

To determine if the increase of mitochondrial ROS in Tat‐treated cells was due to an increase in mitochondrial number, we measured the overall fluorescence intensity of MitoTracker. Tat protein causes a decrease in MitoTracker Green, indicating that Tat increased total ROS and mitochondrial ROS without increasing the mitochondrial number (Figure 1H).

Ca^2+^ signaling regulates mitochondrial bioenergetics and ROS production.^31^ Interestingly, both functions are under the control of MAMs.^32^, ^33^ Further, IP_3_ binds to its receptors (IP_3_Rs) causing the release of Ca^2+^ from ER stores.^34^ Briefly, IP_3_ is very reactive with IP_3_Rs, caged‐IP3 can be loaded into the cells where it can be uncaged by exposing the cells to UV light at the desired time so Ca^2+^ localization can be visualized. Therefore, we assessed the effect of Tat on calcium transfer between the ER and mitochondria in response to IP_3_ using Tat‐treated LUHMES. We treated LUHMES cells with Ca^2+^ indicator dyes that localize to the cytoplasm (Fura2) and mitochondria (Rhod2), respectively. Following the addition of CaCl_2_ and IP_3_ uncaging, we measured the fluorescence. The basal cytoplasmic and mitochondrial calcium levels (Rhod2) were higher in Tat‐treated than in control cells (Figure 1I), which corroborates previous reports.^9^

In response to CaCl_2_ (Ca^2+^‐induced), we did not observe any significant difference in mitochondrial calcium (Rhod2) between the control and Tat‐treated cells. However, the expression level of cytoplasmic Ca^2+^ was higher in Tat‐treated than in the control cells.

Interestingly, Ca^2+^ expression levels decreased in mitochondria of Tat‐treated cells compared to the mock untreated after IP_3_ was uncaged (IP3‐induced) and Ca^2+^ was released from ER stores (Figure 1I). We concluded that even though Ca^2+^ was released from the ER, mitochondria failed to take additional Ca^2+^, so it remained in the cytoplasm, as indicated by the slight increase in cytoplasmic Ca^2+^ (Fura 2). These results demonstrated that Ca^2+^ dynamics, regulated by MAMs, are altered in the presence of Tat.

Tat protein is known to increase glutamate exocytosis and glutamate excitotoxicity in neurons.^35^ Glutamate excitotoxicity was linked to neurodegenerative diseases, and the glutamatergic system has been suggested to be a target of HAND.^36^ Therefore, we measured the expression levels of glutamate in LUHMES treated with Tat protein. Metabolic analysis assay that Tat increased intracellular glutamate concentration in Tat‐treated cells (Figure 1J). Similarly, increased extracellular glutamate in Tat‐treated cells was also observed (Figure 1K). These results indicate that Tat alters the communication between the ER and the mitochondria.

### 3.2 Tat protein alters the expression levels of MAM‐associated proteins

**FIGURE 2:**
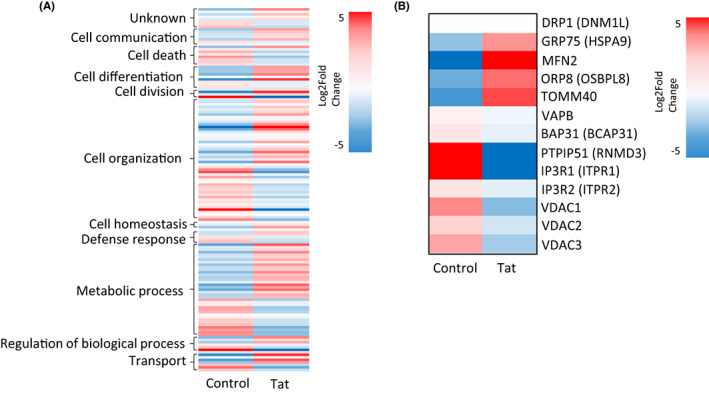
Proteomics analysis displayed as Heatmaps. Heatmaps representing differentially expressed proteins from proteomics analysis organized by (A) cellular functional pathway and (B) association with MAMs between control SH‐SY5Y cells and Tat‐treated cells. Colors represent Log2fold change.

To further validate the ability of Tat to disrupt MAMs, we conducted a proteomic analysis of global protein expression using differentiated SH‐SY5Y cells treated with 100 ng/ml of Tat or PBS for 24 h. Pathway analysis of the most differentially expressed proteins in Tat‐treated cells revealed that many play a role in cell organization, cell homeostasis, and transport, all of which are regulated by MAMs^33^, ^37^ (Figure 2A). When analyzing the data further, we observed that major MAM‐associated proteins are also differentially expressed in Tat‐treated cells compared to control cells (Figure 2B). These results suggest that Tat deregulates the neurons through a mechanism that involve MAMs.

### 3.3 Tat protein deregulates the interaction between MAMs tethering proteins in vitro

**FIGURE 3:**
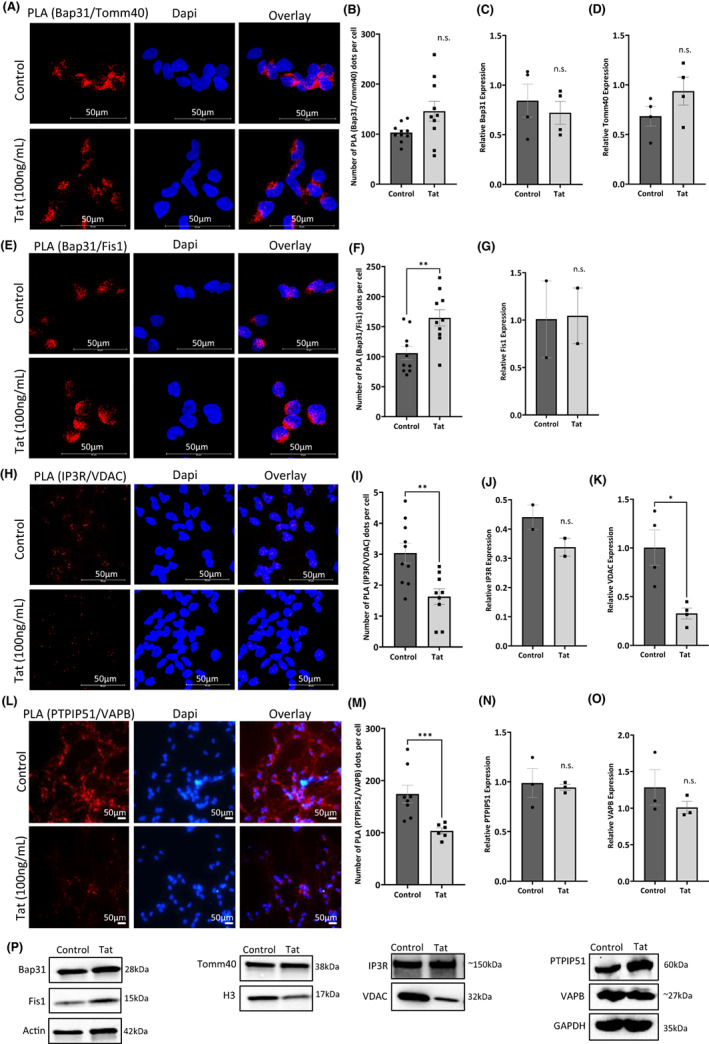
Expression and interaction between MAM tethering proteins in the presence of Tat. Representative images of PLA for the interaction between MAM tethering proteins (A) Bap31 and Tomm40, (E) Bap31 and Fis1, (H) IP3R and VDAC, and (L) PTPIP51 and VAPB in LUHMES cells in the absence and presence of Tat. Red dots indicate individual interactions. DAPI staining indicates individual nuclei. Quantification of assays is represented by bar graphs (B, F, I, and M). Western blot analyses (P) and quantification for the whole cell expression of Bap31 (C), Tomm40 (D), Fis1 (G), IP3R (J), VDAC (K), PTPIP51 (N), and VAPB (O). (NS = not significant; **p < 0.01). Error bars are shown as S.E.M.

Several proteins play a role in MAM maintenance and communication between the ER and mitochondria. These include BAP31, IP3R, and VAPB on the ER and Tomm40, Fis1, RMDN3/PTPIP51, and VDAC on mitochondria.^10^ Therefore, we sought to determine whether Tat protein alters their interactions. We performed a Duolink proximity ligation assay (PLA) using differentiated LUHMES treated with 100 ng/ml of recombinant Tat protein or PBS for 24 h. We observed the interaction between the above tethering proteins (Figure 3A, E, H, L). These proteins tether the ER and mitochondria and regulate critical MAM‐associated functions.^10^ Quantification of the PLA shows that Tat significantly increased the interaction between Bap31 and Fis1, while it decreased the interaction between IP3R and VDAC as well as VAPB and PTPIP51 (Figure 3F, I, M). The interaction between Bap31 and Tomm40 was not significantly affected by the addition of Tat protein despite the slight increase (Figure 3B). These results led us to conclude that Tat protein alters the interactions between key MAM tethering proteins. To assess whether these disrupted associations are due to changes in protein expression levels, we performed western blots using whole‐cell lysates (Figure 3P) (original blots are presented as supplementary data) and the quantifications are presented in Figure 3C, D, G, J, K, N, O. Tat protein did not affect the expression levels of these proteins of these tethering proteins, except VDAC, suggesting that Tat is using another mechanism than protein expression levels.

### 3.4 Tat protein prevents PTPIP51‐VAPB association in HIV‐Tg Rats

**FIGURE 4:**
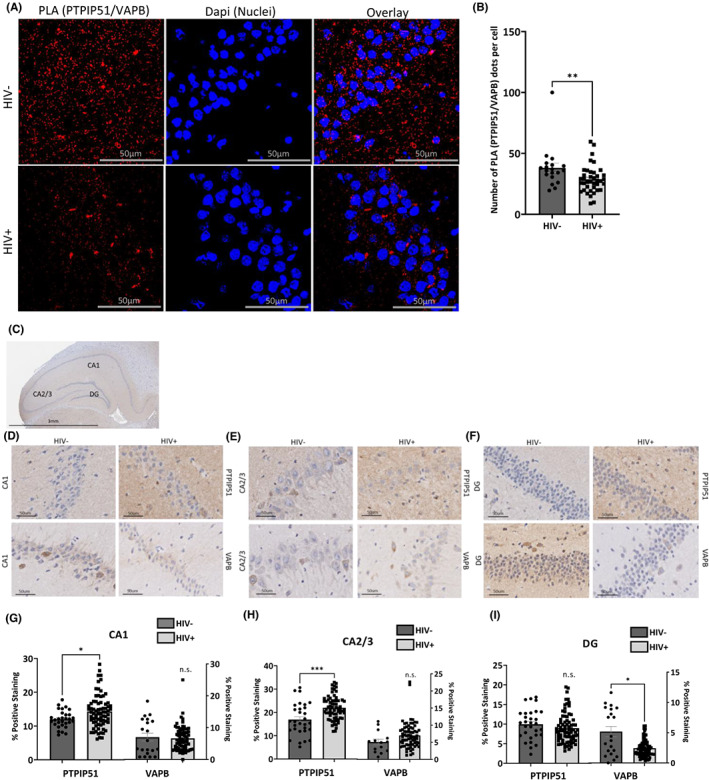
Disrupted PTPIP51 and VAPB interactions and expression in HIV Tg Rats. (A) Representative images of PLA for PTPIP51 and VAPB in hippocampal sections of Tg rat brains. Red dots indicate individual interactions. DAPI staining indicates individual nuclei. (B) Quantification of PLA analysis represented as a bar graph. Representative images of IHC for the expression of PTPIP51 and VAPB in the (D). CA1 region, (E) CA2/3 region, and (F). DG region of the hippocampus as indicated in (C). Quantification of the IHC analysis in the (G) CA1 region, (H). CA2/3 region, and (I). the DG region of the hippocampus represented as bar graphs. Results were judged statistically significant if *p* < 0.05 by analysis of variance. (NS = not significant; *p < 0.05; ***p < 0.001). Error bars are shown as S.E.M.

PTPIP51 is among the MAMs proteins deregulated by the addition of Tat protein. Per the literature, PTPIP51 plays a role in memory formation, and its interaction with its MAM tethering partner, VAPB, has been shown to regulate synaptic activity.^38^, ^39^ To validate our in vitro data, we sought to examine the PTPIP51‐VAPB association in vivo using HIV‐transgenic (tg) rats. We performed a PLA using brain tissue prepared from HIV‐tg rats (Figure 4A). As shown in Figure 4A, B, we observed a significant decrease in the interaction between PTPIP51 and VAPB in HIV‐tg rat hippocampi compared to the control (Figure 4B).

We also examined expression levels of these proteins in HIV‐tg rats' brain tissues using an immunohistochemistry assay (Figure 4C). Expression levels of PTPIP51 protein significantly increased in the CA1 and CA2/3 regions (Figure 4D, E) but decreased in the DG area (Figure 4F). The changes in VAPB protein expression levels were not significant except for their decrease in the DG region (Figure 4F). The quantification of these changes is displayed as bar graphs in Figure 4G‐I. These results confirmed our in vitro data and suggested that the expression of PTPIP51 and VAPB is region‐specific.

### 3.5 The expression of PTPIP51 and VAPB proteins decreases in the MAM fraction

**FIGURE 5:**
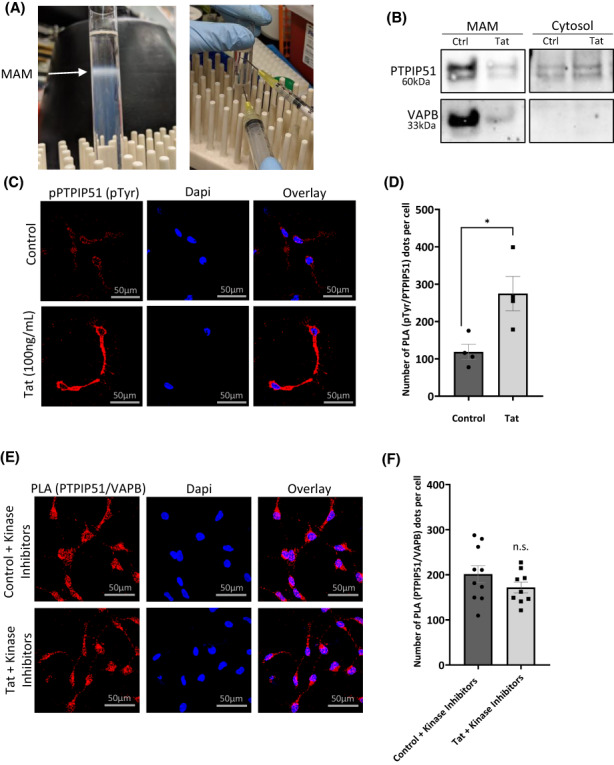
Altered expression in the MAM fraction and PTPIP51 phosphorylation in the presence of TAT. (A) Isolation of MAMs (white ring). (B) Western blot analysis of the expression of PTPIP51 and VAPB in different fractions of control SH‐SY5Y cells and those treated with Tat. (C) Representative images of PLA for tyrosine phosphorylated PTPIP51 (pPTPIP51) in LUHMES cells in the absence and presence of Tat. Red dots indicate phospho‐tyrosine residues. DAPI staining indicates individual nuclei. (D) Quantification of the PLA for pPTPIP51 represented as a bar graph. (E) Representative images of PLA for PTPIP51 and VAPB in LUHMES cells treated with kinase inhibitors (gefitinib, dasatinib, and RpcAMPs) for 4 h in the absence and presence of Tat. Red dots indicate individual interactions. DAPI staining indicates individual nuclei. (F) Quantification of the number of PTPIP51 and VAPB interactions is represented as a bar graph. N.S = not significant, *p < 0.05. Error bars are shown as S.E.M.

Next, we assess the expression levels of PTPIP41 and VAPB proteins in the MAM fraction. LUHMES cells were differentiated and then treated with 100 ng/ml of recombinant Tat protein or PBS. Using a Percoll/sucrose gradient, the cells were fractioned, and the cytosolic and MAM extracts were collected (Figure 5A) and subjected to Western blot analysis using anti‐PTPIP51 and anti‐VAPB antibodies. Expression levels of both proteins significantly decreased in the MAM, but not in the cytosolic fraction, in Tat‐treated cells compared to the mock untreated (Figure 5B).

### 3.6 Tat protein promotes the phosphorylation of PTPIP51 protein

The activity of PTPIP51 depends on its phosphorylation status.^29^, ^40^ For instance, phosphorylated PTPIP51 (pTPTPI51) binds to 14–3‐3 and Raf‐1 proteins in the cytosol and affects the mitogen‐activated protein kinase (MAPK) pathway.^41^ Subcellular localization and association of PTPIP51 with other proteins outside the mitochondria also depend on its phosphorylation on tyrosine residue 176.^40^

To validate this observation, we examined the phosphorylation status of PTPIP51 protein in Tat‐treated cells using a PLA assay using anti‐PTPIP51 and anti‐pTyr antibodies (Figure 5C). Indeed, the presence of Tat protein significantly increases the phosphorylation status of PTPIP51 protein compared to the mock untreated (Figure 5C, D). We concluded that the Tat protein promotes the phosphorylation of PTPIP51. Tat protein also forces PTPIP51 protein to change its subcellular localization, thus preventing PTPIP51 functional association with VAPB causing mitochondrial stress and deregulation.

### 3.7 Tyrosine inhibitors restore PTPIP51‐VAPB association

When PTPIP51 is phosphorylated, it does not localize to the mitochondria and thus cannot interact with its binding partners in the MAM. The PTPIP51 protein is phosphorylated on tyrosine residues by the Lyn and Src families of kinases. To prevent the phosphorylation of PTPIP51 protein on tyrosine residues, several inhibitors were developed. These included Dasatinib (tyrosine kinase inhibitor), Gefitinib (EGFR inhibitor), and Rp‐cAMPS (cAMP‐dependent kinases inhibitor).^12^, ^29^ Our data regarding the phosphorylation of PTPIP51 protein in Tat‐treated cells gave us the rationale to determine whether the addition of the inhibitors neutralizes the effect of Tat. Differentiated LUHMES cells were treated with the inhibitors before adding 100 ng/ml of recombinant Tat protein. After 24 h, the cells were subjected to PLA assay to assess the PTPIP51‐VAPB association. As shown in Figure 5E, tyrosine inhibitors neutralized the Tat protein's effect and allowed PTPIP51‐VAPB association. Figure 5F represents the quantification of PLA that remained unchanged in Tat‐treated cells compared to the mock untreated.

## 4 DISCUSSION

**FIGURE 6:**
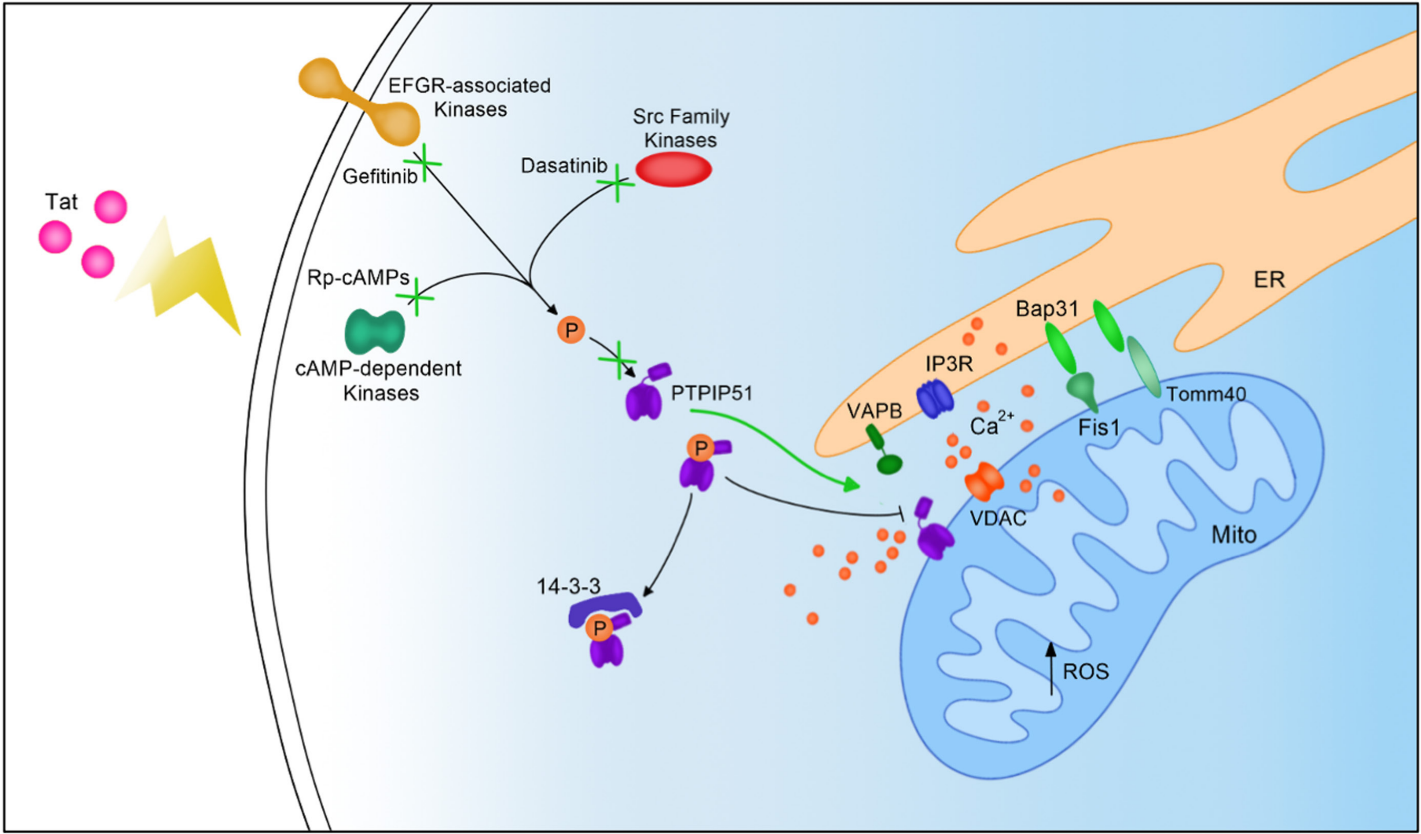
Schematic illustrating the effect of Tat on MAMs in neurons. Tat affects the interaction between MAM tethering proteins IP3R and VDAC, Bap31 and Fis1, Bap31 and Tomm40, and PTPIP51 and VDAC. The affected MAM tethering leads to dysregulated calcium transfer between the ER and mitochondria and increased ROS. Tat also affects PTPIP51 phosphorylation and thus its localization to MAMs. Kinase inhibitors gefitinib, dasatinib, and Rp‐cAMPs together can block PTPIP51 tyrosine phosphorylation even in the presence of Tat and can lead to PTPIP51 and VAPB interactions.

Mitochondrial‐associated ER membranes (MAMs) control many cellular functions, including calcium and lipid exchange, intracellular trafficking, and mitochondrial biogenesis.^19^ We showed the disruption of some of these MAMs' functions in cells treated with HIV‐1 Tat protein (Figure 6). Interestingly, these functions were also disrupted in HIV‐infected patients suffering from HIV‐associated neurocognitive disorders (HAND).^4^, ^9^, ^22^, ^42^ For instance, we demonstrated the ability of Tat protein to decrease the maximal respiration and spare capacity and to increase Ca^2+^ influx and ROS accumulation, which corroborates previous data.^8^, ^42^, ^43^ ROS is known to be regulated in part by MAMs.^44^ Additionally, we demonstrated that Tat protein disrupts the calcium signaling between the organelles mainly because of the decrease in PTPIP51 and VAPB association. This decreased association is due to the phosphorylation and change in subcellular localization of PTPIP51 on tyrosine residue(s) in Tat‐treated cells (Figure 6).

The role of MAMs in neurodegenerative diseases has been established.^45^ For instance, the breaking of PTPIP51‐VAPB tethers contributes to the development of frontotemporal dementia/amyotrophic lateral sclerosis (FTD/ALS).^46^ Also, staining techniques revealed the loss of VAPB‐PTPIP51 interaction in the cortex of Alzheimer's patients.^47^ However, the status of MAMs tethering pair in cells infected or affected by viral infection remains understudied and unclear. Other than a couple of reports, it is hard to find any study linking MAMs to viruses,^17^, ^48^ which makes our study novel and significant. Likewise, while the functions of PTPIP51 were established (autophagy, memory, and synaptic regulation),^16^, ^38^, ^39^ its association with viruses has never been examined.

We focused our study on the interaction between VAPB (ER) and PTPIP51 (mitochondria) proteins. The loss of interaction between the two proteins is not unprecedented. For example, the PTPIP51‐VAPB interaction plays a role in synaptic plasticity, and losing this association decreases synaptic activity.^39^ Further, studies showed that the PTPIP51‐VAPB interaction is mediated by the FFAT‐like (two phenylalanines [FF] in an acidic tract) motif of PTPIP51 and the MSP domain of VAPB.^49^ However, this interaction can be easily disturbed if PTPIP51 is phosphorylated on Tyr176. Furthermore, interaction of the TDP‐43 protein with VAPB causes the loss of PTPIP51‐VAPB association that contributes to the development of ALS/FTD.^50^ Like HAND, ALS/FTD is associated with cognitive impairment and gait problems.

PTPIP51 interactions and phosphorylation have been mainly examined in cancer pathology.^12^, ^51^ Other studies performed on keratinocytes showed increased tyrosine phosphorylation of PTPIP51 allowing its interaction with 14–3‐3b and Raf1.^29^, ^51^ Further, increased expression levels of phosphorylated PTPIP51 on Tyr176 in breast cancer cells were observed.^52^ Also, PTPIP51 has been shown to play the role of tumor suppressor in non‐small cell lung cancer (NSCLC) through its physical interaction with PTEN.^53^ These results validate our data regarding phosphorylation and cytoplasmic expression levels of PTPIP51.

The interaction of PTPIP51 with cellular factors modifies its functions. For example, the EGF receptor physically interacts and inhibits PTPIP51 function in glioblastoma cells.^54^ While Gefitinib, an EGFR inhibitor, restores PTPIP51 expression and function. These results also corroborate our data shown in Figure 5D, where we showed that the addition of Gefitinib along with other inhibitors neutralized the Tat effect. Overexpressed and unphosphorylated PTPIP51 was also shown to interact with oxysterol‐binding protein‐related proteins 5 and 8 (ORP5/8) at MAMs to facilitate phospholipid transfer between the ER and mitochondria.^55^ Like PTPIP51, VAPB was also shown to be involved in lipid trafficking through its interaction with ACBD5 protein, which is controlled by GSK3β.^56^ These data support our findings that loss of PTPIP51 expression level and phosphorylation in Tat‐treated cells renders the protein nonfunctional.

As mentioned above, the effect of HIV‐1 proteins on MAMs remains unexplored; however, a study investigating the HIV‐1 Vpr protein and mitochondrial dysfunction showed that Vpr localizes and integrates into the outer membrane of mitochondria and the ER.^48^ The authors also showed that Vpr disrupts the expression of mitofusin 2 (Mfn2), a MAM‐associated protein. Therefore, future studies must be performed to determine the localization and protein interactions with Tat in neurons to elucidate a possible direct mechanism of MAM dysregulation. Further, it is fundamental to note that we did not observe a consistent decrease in all MAM tethering protein interactions but rather an increase in Bap31 and Fis1 interactions. Therefore, it appears that Tat protein affects MAM‐tethering proteins differently and that the deregulation of MAMs is critical in pathology rather than an overall up or downregulation.

For future studies, we will also examine the direct link between the MAM and HAND in vivo using organoids and animal model, as well the impact of PTPIP51‐VAPB association on glycolysis and mitochondrial bioenergetics, mitophagy, and synaptic plasticity,^57^, ^58^, ^59^, ^60^ since disruption of these functions can contribute to HAND development and premature brain aging.

Overall, this report illuminates multiple potential targets for future studies in HAND pathology, like PTPIP51‐VAPB interaction, PTPIP51 phosphorylation/localization, identification of the kinase involved, and the phosphorylated site. Keep in mind that MAMs remain a novel area of interest in studying aging and neurocognitive disorders, especially HAND pathology.

## AUTHOR CONTRIBUTIONS

Sterling P Arjona and Charles NS Allen designed, performed the studies, and wrote the manuscript. Maryline Santerre provided technical assistance and reagents. Scott Gross and Jonathan Soboloff helped with calcium experiments and data analysis. Jonathan Soboloff edits the manuscript. Rosemarie Booze provided transgenic animals. Bassel E Sawaya directed and supervised the work.

## FUNDING INFORMATION

This work is supported by an NIH‐NIA grant AG054411 and by previous NIH grants NS076402 and MH093331 awarded to BES.

## CONFLICT OF INTEREST

The authors declare that the research was performed in absence of any financial relationships and has no potential conflict of interest.

## Supporting information


Appendix S1

## Data Availability

All processed data are included in this manuscript. Raw data, further information, or reagents contained within the manuscript are available upon request from the corresponding author, Bassel E Sawaya, sawaya@temple.edu.
