# Supplementary material for: Disruption of Mitochondrial‐associated ER membranes by HIV‐1 tat protein contributes to premature brain aging
Source: CNS Neurosci Ther. 2022 Nov 23;29(1):365–77. doi: 10.1111/cns.14011 (PMC9804058; doi:10.1111/cns.14011)

Full Unedited gel/blots presented in Fig 3Q

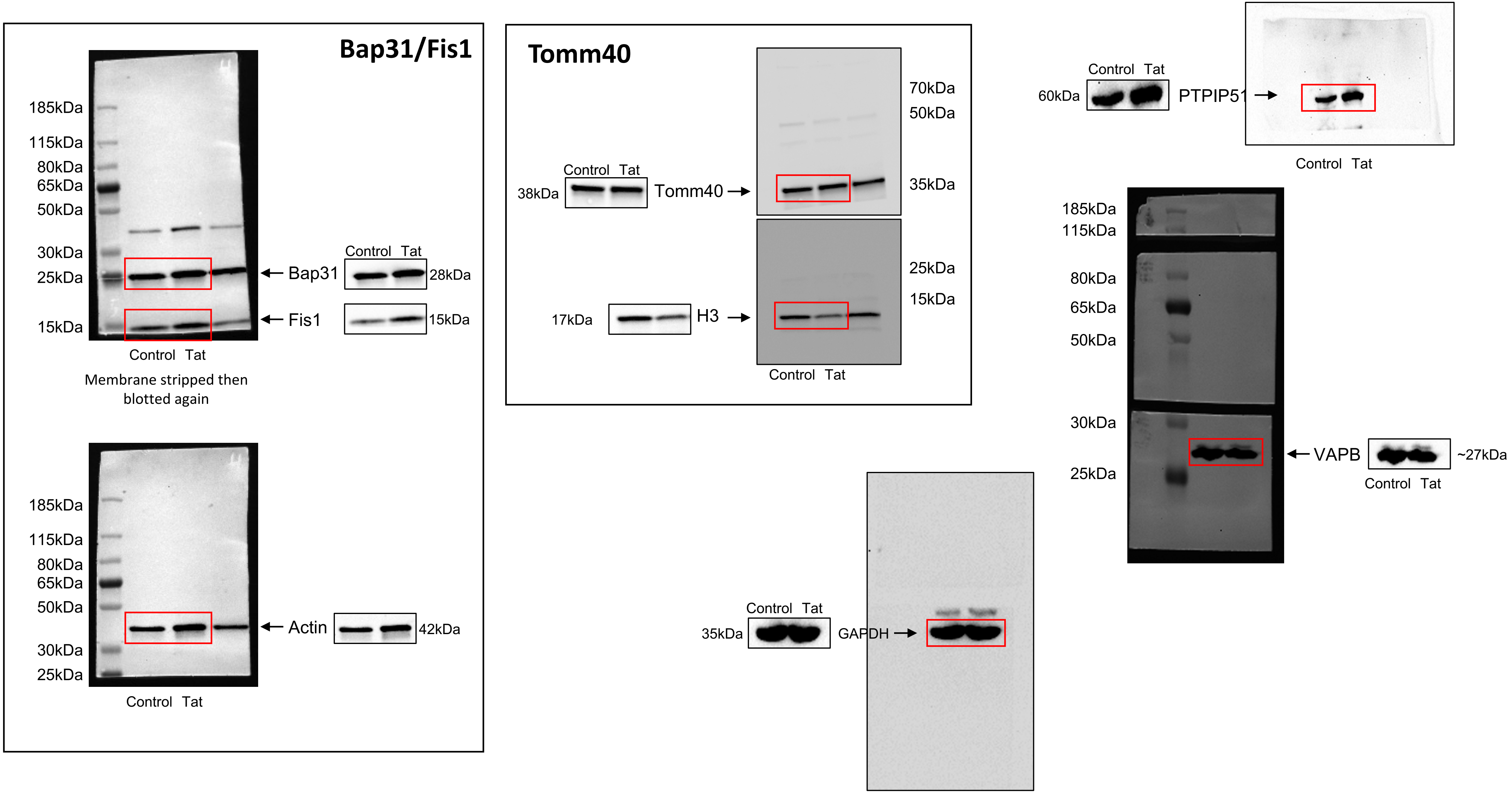

Full Unedited gel/blots presented in Fig 3Q IP3R/VDAC

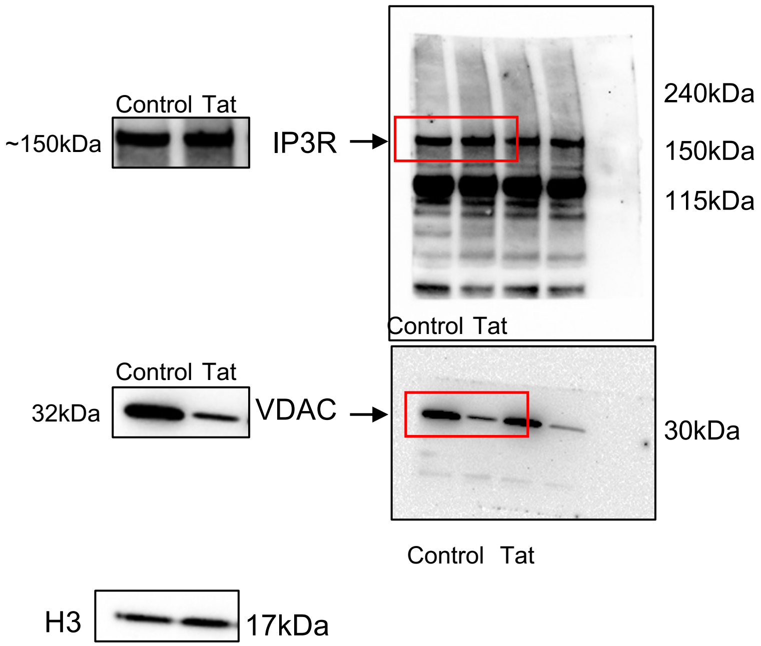

We removed H3 because we could not find the original on the machine (maybe inadvertently deleted)

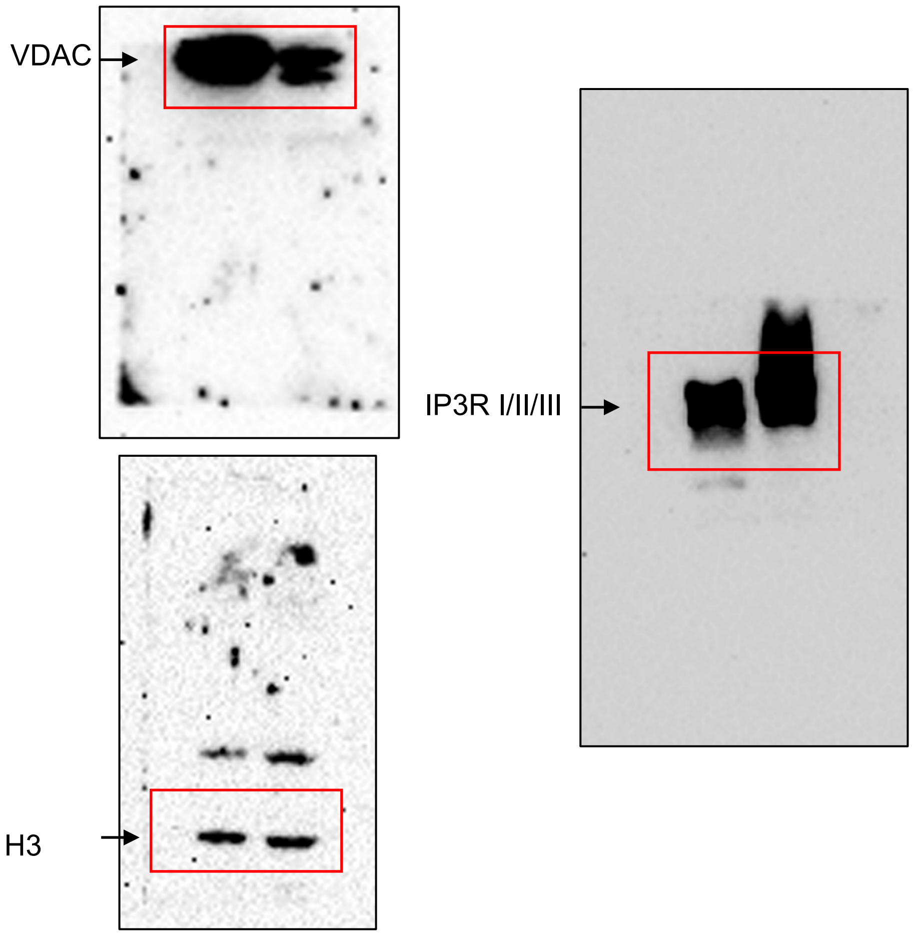

Another Western IP3R/VDAC

Full Unedited gel/blots presented in Fig 5B

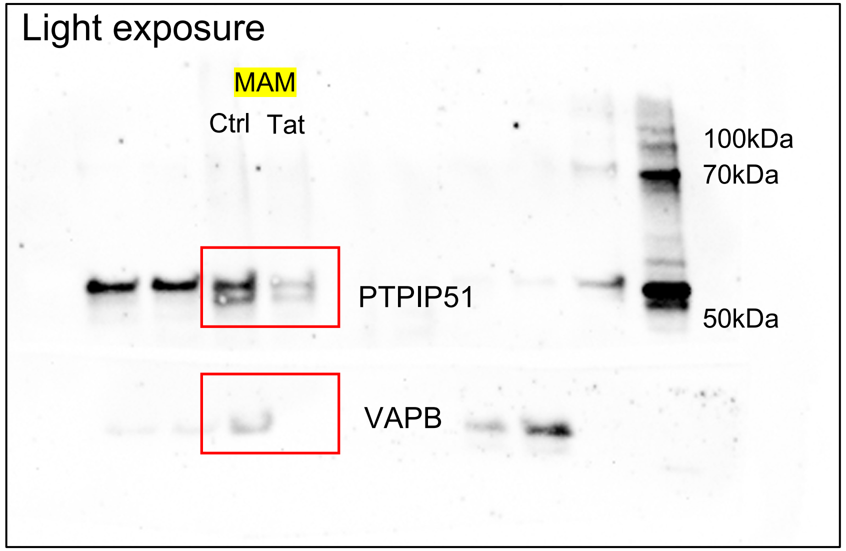

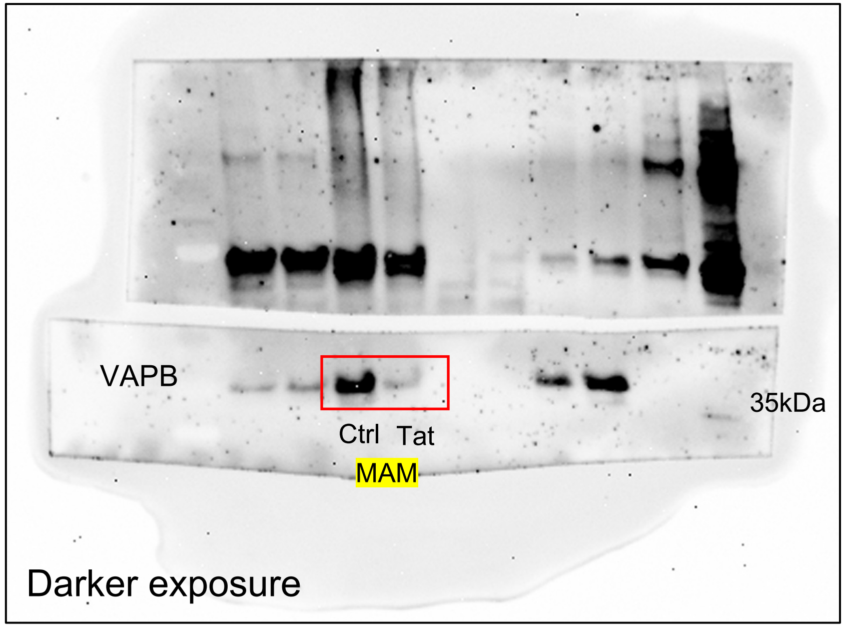

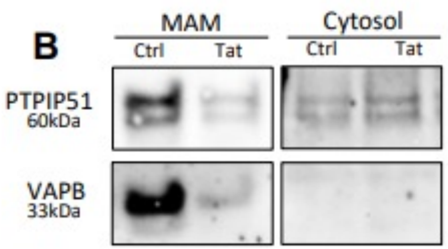

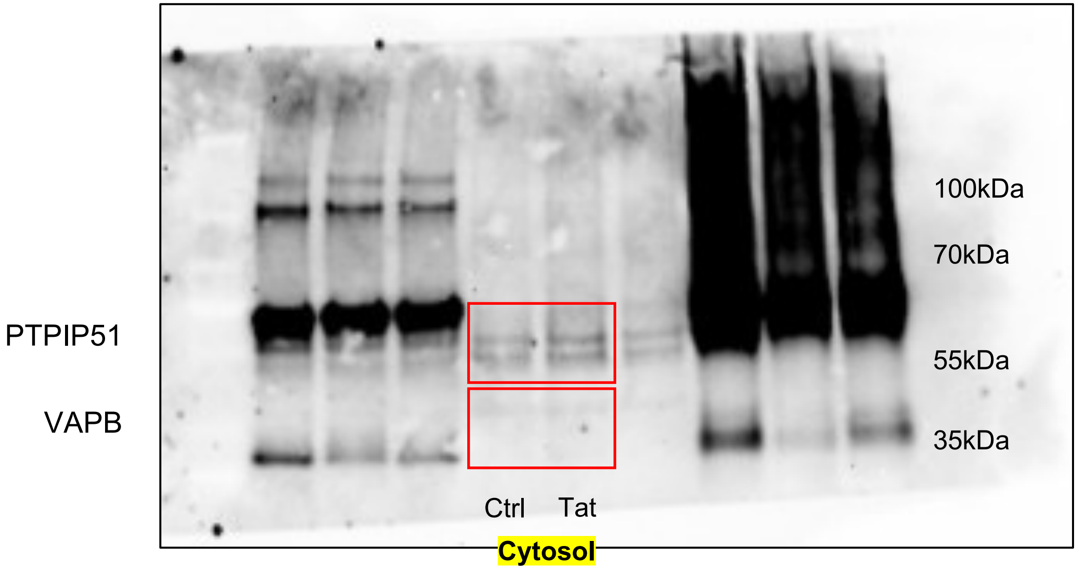

Supplement: Supplementary file 1 — Appendix S1 [file CNS-29-365-s001.pdf]
